# A randomised controlled trial of a lengthened and multi-disciplinary consultation model in a socially deprived community: a study protocol

**DOI:** 10.1186/1471-2296-8-38

**Published:** 2007-06-28

**Authors:** David L Whitford, Wai-Sun Chan

**Affiliations:** 1 Family and Community Medicine, Royal College of Surgeons in Ireland-Medical University of Bahrain, PO Box 15503, Adliya, Kingdom of Bahrain; 2 Department of General Practice, Royal College of Surgeons in Ireland, 121 St Stephens Green, Dublin 2, Ireland

## Abstract

**Background:**

There has been little development of the general practice consultation over the years, and many aspects of the present consultation do not serve communities with multiple health and social problems well. Many of the problems presenting to general practitioners in socio-economically disadvantaged areas are not amenable to a purely medical solution, and would particularly benefit from a multidisciplinary approach. Socio-economic deprivation is also associated with those very factors (more psychosocial problems, greater need for health promotion, more chronic diseases, more need for patient enablement) that longer consultations have been shown to address. This paper describes our study protocol, which aims to evaluate whether a lengthened multidisciplinary primary care team consultation with families in a socially deprived area can improve the psychological health of mothers in the families.

**Methods/Design:**

In a randomised controlled trial, families with a history of social problems, substance misuse or depression are randomly allocated to an intervention or control group. The study is based in three general practices in a highly deprived area of North Dublin. Primary health care teams will be trained in conducting a multidisciplinary lengthened consultation. Families in the intervention group will participate in the new style multidisciplinary consultation. Outcomes of families receiving the intervention will be compared to the control group who will receive only usual general practitioner care. The primary outcome is the psychological health of mothers of the families and secondary outcomes include general health status, quality of life measures and health service usage.

**Discussion:**

The main aim of this study is to evaluate the effectiveness of a lengthened multidisciplinary team consultation in primary care. The embedded nature of this study in general practices in a highly deprived area ensures generalisability to other deprived communities, but more particularly it promises relevance to primary care.

**Trial registration:**

Current Controlled Trials ISRCTN70578736

## Background

### The impact of socio-economic deprivation

There is little doubt that individuals living in areas of socio-economic deprivation suffer poorer health. [1] In 1980 the Research Working Group chaired by Sir Douglas Black produced an authoritative report documenting inequalities in health in Britain. [2] This stimulated a widespread response and since then much more evidence has accumulated about health inequalities in many different countries, [1] including Ireland. [3] In particular, both standardised mortality rates [4] and infant mortality rates [5] are correlated with socio-economic deprivation in Ireland. The all-cause mortality rate on the island of Ireland in the lowest occupational class is 100–200% higher than in the highest occupational group. [6] There is also evidence that morbidity is highest in general practices serving socio-economically deprived areas. This includes psychiatric morbidity, [7] cardiovascular disease,[8] diabetes, [9] cerebrovascular disease, [10] and psychological distress. [11] Inequalities in health are often compounded by inequalities in access to health care. In Dublin, for example, there is evidence that general practices are heavily concentrated in more wealthy areas. [12] Patients from deprived areas are also more likely to have higher consultation rates [13,14] and prescribing costs. [15] Individuals living in these areas have a higher need and use of social services. [16]

The National Health Strategy in Ireland *'Quality and Fairness' *has equity as one of its fundamental principles:

"Equity will be central to developing policies (i) to reduce the difference in health status currently running across the social spectrum in Ireland; and (ii) to ensure equitable access to services based on need". [17]

However, little of a concrete nature has been put in place to address this imbalance between health inequalities and access to health services. Primary and secondary care services are configured to give advantage to those with the least health need. There is little evidence from the literature in Ireland or the UK of attempts to reconfigure primary care in socio-economically deprived areas in order to address health inequalities.

### Consultation length

There have been several review articles of the impact of longer consultation length. [18-20] These conclude that longer consultation length includes more key elements of the consultation, particularly health promotion, health education, preventive measures, improved recognition and handling of psychosocial problems, fewer prescriptions, improved chronic disease management, and better patient enablement. Longer consultations are also associated with greater patient satisfaction and reduced doctor stress. Socio-economic deprivation is associated with those very factors (more psychosocial problems, greater need for health promotion, more chronic diseases, more need for patient enablement) that longer consultations have been shown to address. However, one study concluded that 'increasing socioeconomic deprivation was associated with higher prevalence of psychological distress and shorter consultations. This provides further evidence to support Tudor Hart's 'inverse care law' and has implications for the resourcing of primary care in deprived areas.' [21]

### Multidisciplinary working

Many of the problems presenting to general practitioners (GPs) in socio-economically disadvantaged areas are not amenable to a purely medical solution, and would particularly benefit from a multidisciplinary approach. There are few examples of adopting a multidisciplinary consultation in primary care. One study of a multidisciplinary consultation in Israel reported creating a multidisciplinary clinic in the community for frequent attenders. The intervention consisted of a comprehensive bio-psychosocial consultation where life history and medical symptoms were woven together into a new narrative. The intervention also included pharmacological treatment and short-term psychological interventions. Consultations and treatment costs fell by two thirds after one year. They concluded that the integrated approach of the clinic satisfied at least three needs: of the patient, of the referring physician and of the health maintenance organization. [22]

### Hypothesis

This study sets out to evaluate an alternative way of delivering primary care in a socio-economically disadvantaged community. The intervention incorporates both a multidisciplinary team approach to the consultation and a lengthened consultation time.

The null hypothesis is that implementation of a lengthened and multidisciplinary primary care consultation will have no impact upon the psychological health of mothers in terms of anxiety and depression as measured by the Hospital Anxiety and Depression Scale (HADS).

### Objectives

Our primary objective is to evaluate the effectiveness of a lengthened multi-disciplinary consultation in primary care with socially deprived families on the psychological health of the mothers in the family as compared with normal care. Our secondary objective is to assess the impact of the intervention on health status and quality of life; on health service use; and on health promotion outcomes including smoking, alcohol and substance use.

## Methods/Design

The study is a randomised, controlled, intervention trial where families (with at least one child under the age of 16 years) with a history of social problems, substance misuse or depression are allocated to one of two groups:

• Intervention group where families will receive a lengthened multidisciplinary consultation in primary care.

• Control group where families will receive usual GP care only.

### Ballymun Primary Care Implementation Project

Ballymun is a suburb of North Dublin, Ireland covering an area ofapproximately two square miles. It had a population of 16,568 at the 1996 census, with an atypical age profile. Thereis a disproportionately high proportion of females aged 20 to 49 and low proportion of over 65 year olds (3.1% compared to 11.4% in the State). There was a rise in lone parent families from 28% in 1991 to 37% in 1996. Almost half of all children in Ballymun are reared in lone parent families. Ballymun is an area of extreme deprivation with a HASSE deprivation index of 10 (equivalent to the highest deprivation ranking). This index measures educational attainment, housing and employment status. [23] The area is characterised by educational and economic disadvantage highlighted by the following points:

• High levels of early school leaving.

• Widespread and persisting school truanting.

• Poor levels of educational attainment.

• Low levels of transfer to third level education.

• Social welfare is the sole income of 71% of the population in Council accommodation.

• Very high unemployment rates.

As a consequence there are great health and social needs in the community. Health and social services are delivered from the local health centre and from a variety of other facilities in the town centre. Three general practices operate from the health centre and have a sole commitment to Ballymun. These are soon to be relocated into a purpose built Health Care Unit.

The three general practices were successful in becoming one of ten Primary Care Implementation Project under government policy in Ireland. [24] The project proposal incorporated the new Health Care Unit with capacity for the full range of primary care services, an integrated multidisciplinary team approach to primary care, and the development of disease prevention, rehabilitation and social services to complement the existing diagnosis and treatment focus. The multidisciplinary team will include GPs, practice nurses, public health nurses, physiotherapist, occupational therapist, social worker, community psychiatric nurses and dietician.

Ballymun is an ideal place to carry out this trial in view of its status as a Primary Care Implementation Project and its high level of socio-economic deprivation.

### Identification and recruitment of patients

**Figure 1:**
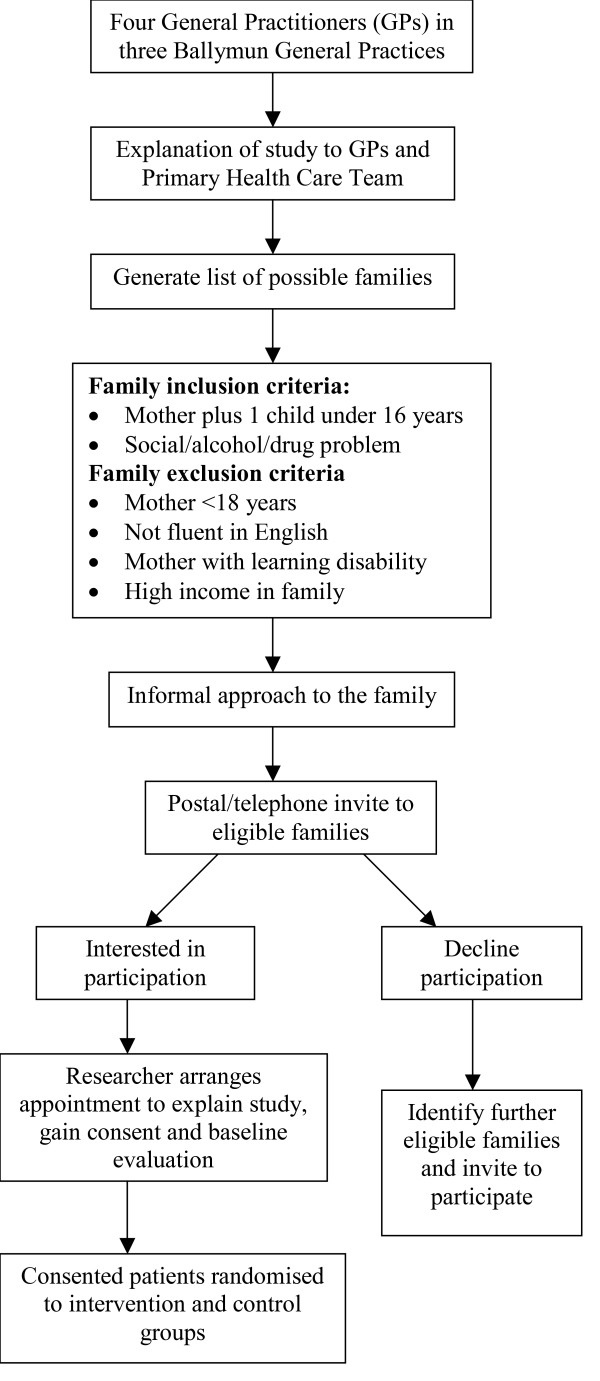
Flowchart of study recruitment process.

Figure 1 shows the recruitment process of patients into the study.

The four participating GPs and the participating primary care team members in the three practices will identify eligible families and make an informal approach to ascertain interest in participation. They will provide lists of eligible families with a history of social problems, substance misuse or depression. Once a list of eligible families has been identified, mothers of the families will be invited to participate in the research by letter and subsequent telephone follow up. A description of the project will be provided with the letter. The letters will be on practice letterhead and signed by the GP. Patients respond by returning the expression of interest form to the research team. A researcher contacts all mothers who express interest in order to arrange an initial face-to-face interview. At this interview the study is fully explained, consent is obtained and baseline data is collected.

### Inclusion and exclusion criteria

All families attending the three general practices in Ballymun are eligible to participate. Families must include a mother and have at least one child under the age of 16 years. The families must also have social and/or mental health and/or alcohol and/or drug problems. Families are excluded if the mother of the family is under 18 years of age; where English is not the first language; where mothers have a learning disability or dementia; or where the family has a high financial income (defined as not being eligible for a GMS card – a means tested access to free medical care in Ireland – €266.50 per week threshold in 2006 for a single parent with dependent children).

### Primary outcome

The primary outcome of this study is the psychological health of mothers in terms of anxiety and depression as measured by the Hospital Anxiety and Depression Scale (HADS). [25] Mothers have been chosen for several reasons. Measuring the health of a 'family' is difficult so it was decided to look for a target member of the family. Mothers are a key axis to the health and health care of families. There is evidence that mothers in deprived settings have poor mental health, and it is suggested that improved well-being of mothers is seen as a key target for improved family health and lifestyle. [26] In addition, 50% of families in the Ballymun area are single parent families and it is likely that the majority of the single parents are mothers. The HADS was also chosen for several reasons. High levels of anxiety and depression are expected in mothers in this area. HADS has been widely used and validated in primary care settings and norms for communities are available. It was therefore decided that it would perform well as a measure in this study.

### Baseline assessment

Baseline data are collected from all study participants using questionnaires, GP computer records, and from the records of multidisciplinary team clinical meetings. Baseline assessment is conducted by a researcher and takes approximately one hour to complete.

Patient written consent is obtained prior to undertaking baseline assessment. Patients' baseline demographic details were taken upon recruitment: Age, marital status, employment status, number of children, and details of living accommodation, smoking status, and alcohol and drug history are noted. Health service use was also assessed by measuring the frequency of participants' GP and practice nurse visits. All patients' consultations with their GPs and practice nurses are held in the practice computer records. This allows the researcher to search for the number of GP and practice nurse attendances in the period 12 months prior to recruitment, and 12 months after. Three questionnaires were administered: Hospital Anxiety and Depression Scale [25] (HADS- a measure of anxiety and depression); SF36v2 [27] (a measure of general health status); and SEIQoL-DW [28] (a measure of quality of life).

### Outcome assessment

Data collected at baseline, including primary and secondary outcome measures, will be collected again at 6 and 12 months post-intervention for both the intervention and control groups. A researcher will collect 6 month and 12 month follow up data by face-to-face interview with the patient. We will also access administrative data sets in relation to health service use.

### Qualitative assessment

A focus group study with patients prior to the commencement of the trial will be carried out to assess views on current primary health care provision in Ballymun and acceptability of a lengthened multidisciplinary consultation. Semi-structured interviews will be carried out at the end of the study with a purposive sample of family participants and with all participating primary care team members. The purpose of this is to assess views of participants towards the intervention.

### Randomisation

**Figure 2:**
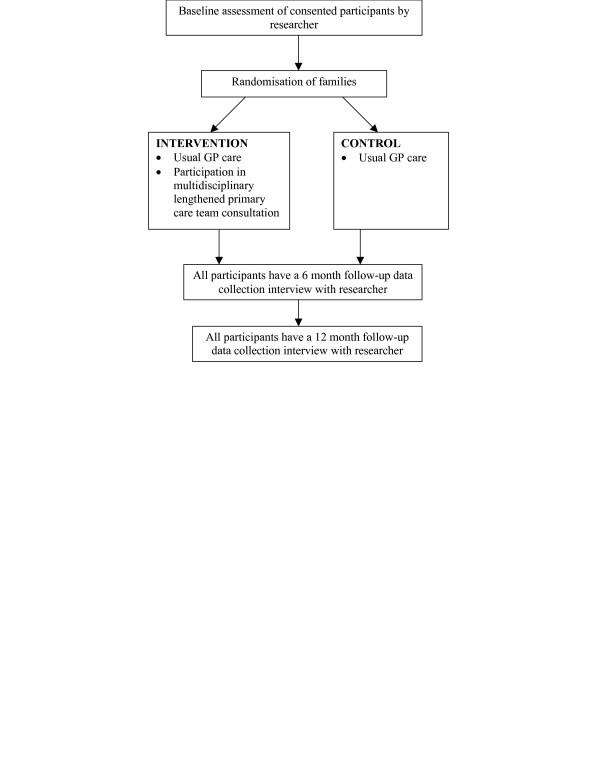
Flowchart of study intervention process.

Families are randomised to either the intervention or control group after all baseline assessments are completed (figure 2). Following randomisation, families are informed by a letter from the principal investigator of whether their family will be in the intervention or control group.

### The intervention

Families in the intervention group will be offered a multidisciplinary consultation with the Primary Health Care Team (PHCT) to assess their health and social needs. The multidisciplinary appointments with intervention patients will be in a phased manner to protect against major service disruption. The four GPs in the study will set aside one three hour session per fortnight each for the study. Intervention families will be presented and discussed openly at the regular Primary Health Care Team meetings in order to gain maximal input and advice. The PHCT will also decide during this meeting who will attend the consultation and arrange a time and date for it. The GP will inform the family, and confirm the time and date for the consultation with the family.

The PHCT members attending the consultation will meet 10 minutes prior to the consultation to discuss the family and appoint one member of the team to lead the consultation process. This member will make introductions, explain the process to the family and open the consultation. The consultation will follow the family's agenda. All areas of health and social functioning will be explored. An appropriate management plan will be made during the consultation and agreed with the family. This will also establish the need for further lengthened consultations with members of the primary care team. A written summary of the consultation should be made for research follow up purposes. Minimum detail should include date and duration of the consultation, team members present in the consultation, and the management plan or follow up agreed from the consultation.

### Quality assurance

The research team will provide assistance to the researcher in the baseline interviews and questionnaire administration to ensure quality of data collection. All general practitioners and primary care team members attended training sessions for the multidisciplinary lengthened consultation, including role plays with simulated patients. Regular feedback on the sessions will occur at primary care team meetings attended by one of the principal investigators (WSC). All primary care members involved in the study will be asked to participate in a semi-structured interview at the conclusion of the study to understand factors influencing variation in implementation of the intervention.

### Sample size calculations

The primary outcome (HADS) is not normally distributed in the population and it is therefore not possible to do a normal sample size calculation as it is not characterised parametrically. The sample size calculation is therefore based on the Mann Whitney statistic. If the variable was normally distributed two groups of 46 families at 90% power and a significance level of 5% would be able to detect a situation in which the chances of performing better in the intervention group rather than the control group would be 75%. A total of 92 families will be required to detect this difference.

### Data collection, monitoring and analysis

Descriptive statistics will be used to summarise patient factors for the two study groups and to compare baseline variables between control and intervention group. Outcomes between study groups will be compared to assess the effect of the intervention. Primary and secondary outcome measures at 6 months and 12 months time frames will be compared between intervention and control groups adjusting for baseline values using an ordinal logistic regression model.

### Trial organization and management

The study has received ethics approval from the Research Ethics Committee of the Royal College of Surgeons in Ireland (Ref REC2004/115). No significant risks to participants are anticipated. As the study is unblinded and low risk a data monitoring committee is unnecessary.

## Discussion

There has been little development of the general practice consultation over the years, and many aspects of the present consultation do not serve communities with multiple health and social problems well. A major strength of this study is that it provides both a new model of the consultation and the opportunity to evaluate it in a randomized controlled trial. This opportunity presents infrequently as many service developments are implemented before adequate evaluation. The study also sets out to recruit a population that is often excluded in other studies. As such, it may contribute towards a reduction in health inequity. The embedded nature of this study in general practices in a highly deprived area ensures generalisability to other deprived communities, but more particularly it promises relevance to primary care.

## Competing interests

The author(s) declare that they have no competing interests.

## Authors' contributions

DLW conceptualised the research. DLW and WSC developed the study design. DLW and WSC oversaw the running of the trial and drafted the protocol.

Both authors read and approved the final manuscript.

## Pre-publication history

The pre-publication history for this paper can be accessed here:


